# Prognostic value of persistent node involvement after neoadjuvant chemotherapy in patients with operable breast cancer

**DOI:** 10.1054/bjoc.2000.1461

**Published:** 2000-11-22

**Authors:** J-Y Pierga, E Mouret, V Diéras, V Laurence, P Beuzeboc, T Dorval, T Palangié, M Jouve, A Vincent-Salomon, S Scholl, J-M Extra, B Asselain, P Pouillart

**Affiliations:** Medical Oncology Department, Biostatistics Department, Pathology Department, Institut Curie, 26 Rue d'Ulm, Paris Cedex 05, 75248, France

**Keywords:** neoadjuvant chemotherapy, breast cancer, pathological nodal metastasis

## Abstract

Neoadjuvant chemotherapy is able to reduce the size of the majority of breast tumours and down-stage axillary-node status. The aim of this study was to assess the prognostic value of persistent node involvement after neoadjuvant chemotherapy. A total of 488 patients with T2–T3, N0–N1 breast cancer treated by neoadjuvant chemotherapy followed by tumour excision and axillary lymph-node dissection between 1981 and 1992 were selected from the Institut Curie database. Median follow-up was 7 years. Overall objective response rate before local treatment was 52% and breast tumour size was reduced in 83% of patients. No pathologic nodal involvement was observed in 46.5% of patients. Patients with ≥ eight positive nodes had a very poor median disease-free survival of only 20 months. Their 10-year disease-free survival rate was 7%, while the 10-year disease-free survival rate for patients with no node involvement was 64%. Median survival for patients with ≥ eight nodes positive was 48 months and the 10-year survival rate was 26% (P < 0.0001). On multivariate analysis, outcome was strongly correlated with pathological nodal status, tumour grade, hormonal receptor status and clinical response of the tumour. In conclusion, patients with extensive nodal involvement after neoadjuvant chemotherapy have a very poor outcome. Second-line treatment should be considered in this population. © 2000 Cancer Research Campaign http://www.bjcancer.com


*British Journal of Cancer* (2000) **83**(11), 1480–1487
© 2000 Cancer Research Campaign
doi: 10.1054/ bjoc.2000.1461, available online at http://www.idealibrary.com on IDEAL®

### Prognostic value of persistent node involvement after neoadjuvant chemotherapy in patients with operable breast cancer

**J-Y Pierga[1], E Mouret[2], V Diéras[1], V Laurence[1], P Beuzeboc[1], T Dorval[1], T Palangié[1], M Jouve[1], A Vincent-Salomon, S Scholl[1], J-M Extra[1], B Asselain[2] and P Pouillart[1]**

[1]Medical Oncology Department; [2]Biostatistics Department; [3]Pathology Department, Institut Curie, 26 Rue d'Ulm, 75248 Paris Cedex 05, France

Summary Neoadjuvant chemotherapy is able to reduce the size of the majority of breast tumours and down-stage axillary-node status. The aim of this study was to assess the prognostic value of persistent node involvement after neoadjuvant chemotherapy. A total of 488 patients with T2–T3, N0–N1 breast cancer treated by neoadjuvant chemotherapy followed by tumour excision and axillary lymph-node dissection between 1981 and 1992 were selected from the Institut Curie database. Median follow-up was 7 years. Overall objective response rate before local treatment was 52% and breast tumour size was reduced in 83% of patients. No pathologic nodal involvement was observed in 46.5% of patients. Patients with ≥ eight positive nodes had a very poor median disease-free survival of only 20 months. Their 10-year disease-free survival rate was 7%, while the 10-year disease-free survival rate for patients with no node involvement was 64%. Median survival for patients with ≥ eight nodes positive was 48 months and the 10-year survival rate was 26% ($P < 0.0001$). On multivariate analysis, outcome was strongly correlated with pathological nodal status, tumour grade, hormonal receptor status and clinical response of the tumour. In conclusion, patients with extensive nodal involvement after neoadjuvant chemotherapy have a very poor outcome. Second-line treatment should be considered in this population. © 2000 Cancer Research Campaign http://www.bjcancer.com

**Keywords**: neoadjuvant chemotherapy; breast cancer; pathological nodal metastasis

The use of neoadjuvant chemotherapy in operable breast cancer may, at least in theory, eliminate early systemic micrometastases, avoid rapid growth of metastases after treatment of the primary site and hopefully prevent emergence of resistant clones (Bhalla and Harris, 1998). The only demonstrated benefit in terms of treatment effects is the achievement of tumour shrinkage, which allows more conservative treatment in some patients (Fisher et al, 1997). Several clinical trials have compared preoperative and postoperative chemotherapy in operable breast cancer, but no significant advantage in terms of long-term survival has been demonstrated to date (Scholl et al, 1994; Semiglazov et al, 1994; Powles et al, 1995; Fisher et al, 1998; Mauriac et al, 1999). The response of breast tumours to preoperative chemotherapy might also be predictive of efficacy of therapy on distant disease and outcome. A possible advantage of primary systemic treatment is to test in vivo tumour response in order to modify treatment or introduce new drugs postoperatively. Finally, the assessment of factors associated with clinical and pathological response is important for prognosis.

The NSABP B-18 trial showed that neoadjuvant chemotherapy was able to reduce the incidence of positive nodes (Fisher et al, 1997). The presence of axillary lymph-node metastases represents the single most important prognostic factor for patients undergoing surgery for primary breast cancer (Haagensen, 1977; Valagussa et al, 1978). The prognosis is also inversely related to the number of involved nodes (Nemoto et al, 1980; Fisher et al, 1983; Carter et al, 1989). Since neoadjuvant chemotherapy reduces node involvement, it could possibly modify the prognostic value of this parameter.

At the Institut Curie, primary radiotherapy for operable breast carcinoma, in order to allow conservative surgery, has been used for decades. Since 1981, neoadjuvant chemotherapy has also been used in large operable breast cancer, prior to local–regional treatment. The aim of this retrospective study was to assess the prognostic value of the persistence of positive axillary nodes after preoperative chemotherapy either by itself or in combination with other prognostic factors.

### PATIENTS AND METHODS

### Patient selection

The present study is a retrospective analysis of the Institut Curie Breast Cancer database. The selection criteria were prior neoadjuvant chemotherapy for operable T2 or T3, N0 or N1 tumours and surgery with axillary dissection. Patients with metastatic, locally advanced or inflammatory cancer were excluded, as were patients with bilateral tumours, prior cancer and male patients. Prognostic factors were assessed in patients with a follow-up greater than 5 years and for whom information was available about pathological axillary-node involvement.

Between 1981 and 1992, 936 patients who received neoadjuvant chemotherapy were registered and selected according to our

*Received 23 March 2000*
*Revised 12 July 2000*
*Accepted 18 July 2000*

*Correspondence to*: J-Y Pierga criteria, except for axillary dissection. Response rates and survival data were assessed in the total population, as a 'reference group'. Axillary dissection was performed in only 507 of these patients. Radiotherapy was proposed as an alternative to surgery. Axillary dissection was performed in only one half of the patients selected in our retrospective study, because of various strategies for local–regional treatment according to patient and/or physician preference, controlled trial arm and tumour response. Radiotherapy alone was predominantly performed in good responders to chemotherapy and/or radiotherapy. In 19 patients, surgery with axillary dissection was performed at the time of local relapse and these patients were excluded from the analysis. Finally, 488 patients were fully eligible for analysis of the prognostic value of persistent node involvement following primary chemotherapy.

Pathological diagnosis and histological grading was performed in all patients on drill biopsy specimens. Steroid receptor levels were assessed by quantitative radioimmunoassay. From 1986 onwards, the cellular S-phase fraction (SPF) was determined in 225 tumours (46%), according to previously described techniques (Remvikos et al, 1993). The cut-off value was 5%, above which the proliferative index was considered to be high.

### Treatment modalities

All patients received a median of four (1–6) cycles of neoadjuvant chemotherapy. From 1983 onwards, chemotherapy consisted of FAC or FEC with adriamycin (doxorubicin) 25 mg $m^{-2}$ day 1 and day 8 or epirubicin 50 mg $m^{-2}$ day 1, cyclophosphamide 500 mg $m^{-2}$ day 1 and day 8, 5-fluorouracil 500 mg $m^{-2}$ day 1, day 3, day 5 and day 8. Fifty nine (12%) of the 488 patients did not receive anthracyclines, but thiotepa at a dose of 10 mg $m^{-2}$ day 1 and day 8, in one arm (CTF) of a randomized trial. Before 1983, i.e. for 32 patients (6.5%), chemotherapy consisted of M2AC with doxorubicin 50 mg $m^{-2}$ day 1, cyclophosphamide 500 mg $m^{-2}$ day 1 and methotrexate 25 mg $m^{-2}$ day 2 and day 9. Chemotherapy was administered intravenously at 28-day intervals or longer depending on bone-marrow recovery. 278 (57%) of the 488 patients were included in three different prospective trials. The data for the rest of the patients were also prospectively registered.

After neoadjuvant chemotherapy and before local-regional treatment, response was assessed by clinical measurements of both the primary tumour and the axillary nodes. Response was scored according to the Eastern Cooperative Oncology Group (ECOG) criteria (Oken et al, 1982). A pathological complete response was characterized as pCR when there was no evidence of residual invasive tumour in the breast or axillary lymph nodes.

Local–regional treatment consisted of surgery with axillary dissection either alone or combined with radiotherapy. Conservative breast treatment consisting of tumourectomy before or following radiotherapy was performed in 236 patients (48.4%). Mastectomy could not be avoided in 252 patients (51.6%) and was associated with radiotherapy in 179 patients (71%). In 166 patients (34%), radiotherapy to the breast was performed before surgery in order to increase the chances of breast preservation. Radiotherapy was delivered at a mean dosage of 54 Gy over 6 weeks to the breast or chest wall and lymph-node areas. Patients with complete or almost complete response received a radiation boost to the tumour bed to achieve a total dose of 75–80 Gy.

In controlled trials, postoperative treatment was not planned. For patients who were not included in controlled trials, adjuvant tamoxifen could be given according to the hormonal status of the tumour.

### Statistical methods

Survival time and disease-free survival time were measured from the date of diagnosis to the date of death or last follow-up. Differences between treatment groups were analysed by Chi-square tests for categorical variables and Student t-test for continuous variables. The survival and response duration curves were determined using a Kaplan – Meier product-limit method (Kaplan and Meier, 1958; Mantel, 1966). Statistical significance between treatment groups was assessed using the log-rank test. Multivariate analysis was carried out to assess the relative influence of prognostic factors on disease-free survival and overall survival, using the Cox proportional hazards model in a forward stepwise procedure (Cox, 1972). Missing values (tumour grade, receptor levels) were coded as separate variables (missing, not missing) and were retained in the model. So the Cox models were done on the whole sample. $P$ values < 0.05 were considered as significant. Statistical analyses were performed by BMDP software (BMDP Statistical Software Inc, Los Angeles CA, USA).

Nodal extent was divided into 0, 1–3, 4–7, and ≥ 8 nodes in order to increase the number of patients in the last group and to permit comparison with a current French multicentric adjuvant trial in poor prognostic breast cancer patients (PEGASE 01).

### RESULTS

### Patient characteristics

Median age was 47 years and 75% of patients were premenopausal (Table 1). Median tumour size was 4.5 cm and 55% of patients had clinical lymph-node involvement. The pathological and laboratory characteristics of the tumours were as follows: 80% of tumours were grade 2 or 3, 87% of tumours were ductal carcinomas, progesterone receptors (PR) were positive in 52% of patients and oestrogen receptors (ER) were positive in 57% of patients, S phase was greater than 5% in 41% of patients. Patients who had received preoperative chemotherapy and radiotherapy had the same proportion of nodal involvement as those treated with preoperative chemotherapy only, particularly for women with ≥ 8 nodes positive (6% vs 7.5%; ns).

### Tumour response to neoadjuvant chemotherapy

Objective clinical response rates immediately prior to local–regional therapy were 52% with only 7% complete responses. Another 31% of patients achieved a minor response. Only 2% of patients presented tumour progression (Table 2). One patient was lost to follow-up before response assessment. Mastectomy was avoided in 48% of the patients in favour of lumpectomy together with radiotherapy. Conservative treatment was performed in a total of 68.2% of the patients who achieved a major response. Conversely, mastectomy was avoided in only 27% of the patients who did not achieve an objective clinical response. Pathological response was assessed and available in the 288 patients not irradiated prior to surgery. The pathological complete response rate was 5% (14 patients) and was significantly correlated with clinical response ($P$ = 0.047). Only one patient classified as a non-clinical responder had a pathological CR. The objective clinical response rate for the complete 'reference group'

© 2000 Cancer Research Campaign

**Table 1** Patient characteristics

| **Patient characteristics** | ***n* (488)** | |
|---|---|---|
| Tumour size | | |
| T2 | 312 | 64% |
| T3 | 176 | 36% |
| Median (range) | 4.5 cm | (2–12 cm) |
| Clinical lymph-node status | | |
| N0–N1a | 219 | 45% |
| N1b | 269 | 55% |
| Age ≤ 40 years | 107 | 21.9% |
| Median (range) | 47 years | (24–71 years) |
| Premenopausal status | 363 | 74.8% |
| SBR Grade | | |
| I | 85 | 20% |
| II | 250 | 59% |
| III | 88 | 21% |
| Histology | | |
| ductal | 420 | 87% |
| lobular | 32 | 6.6% |
| others | 30 | 6.4% |
| missing | 6 | – |
| Pregesterone receptors | | |
| negative | 202 | 47.6% |
| positive | 222 | 52.4% |
| missing | 64 | – |
| Oestrogen receptors | | |
| negative | 165 | 42.8% |
| positive | 220 | 57.2% |
| missing | 103 | – |
| S phase | | |
| ≤ 5% | 132 | 58.6% |
| >5% | 93 | 41.4% |
| missing | 262 | – |

**Table 2** Clinical response evaluated before local–regional treatment

| | ***n* (487)** | **Percentage** | **95% CI** |
|---|---|---|---|
| Complete response | 34 | 7.0% | 4.7– 9.3 |
| Partial response ≥ 50% | 221 | 45.4% | 41–49.8 |
| Minor response < 50% | 152 | 31.2% | 27.1–35.3 |
| Stabilization | 70 | 14.4% | 11.3–17.5 |
| Progression | 10 | 2.0% | 0.8– 3.2 |

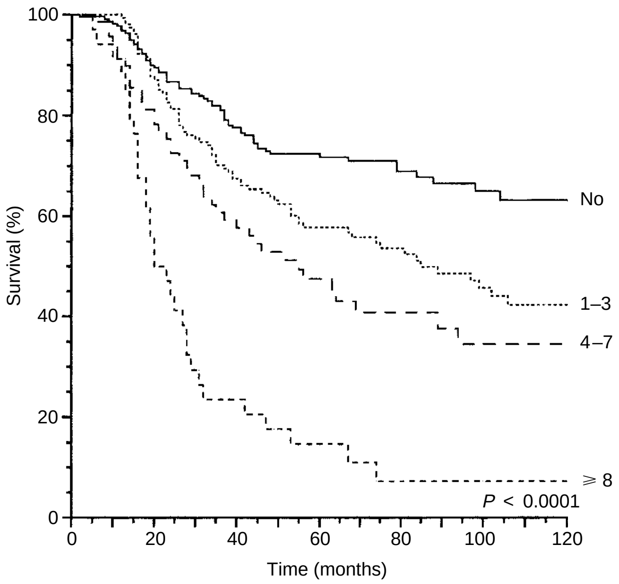

**Figure 1** Disease-free survival according to node involvement ($n$ = 487)

(936 patients), which included the 488 patients analysed in the present report, was 58.3% with 15.5% of complete clinical responses. The proportion of conservative treatment in the entire population was 71.6%.

### Long-term outcome

Median follow-up was 7 years (85 months, range 7–181 months). 157 deaths, 61 local relapses, 191 distant metastases and 215 events have occurred to date. Overall 5-year and 10-year survival rates were 76% (95% CI, 71.8–79.8) and 55% (95% CI, 48.3–60.8), respectively (Table 3). The impact of persistent lymph-node involvement after neoadjuvant chemotherapy on disease-free survival was strongly correlated with the number of positive nodes on axillary dissection (Figure 1, Table 4). Only 15 patients had more than 10 involved nodes. The median disease-free survival was not reached at a median follow-up of 7 years, particularly in patients with no node involvement, who represented 46% of the total population. In contrast, the median disease-free survival for patients with eight or more positive nodes was only 20 months. The 10-year disease-free survival rate was 7% and a similar impact was observed on overall survival (Figure 2, Table 4). The 10-year disease-free survival rate for patients with no node involvement was 64%. Median survival for patients with eight or more positive nodes was 48 months and the 10-year survival rate was 26%. The same analysis was performed in the subgroup of 322 patients who had not received preoperative radiotherapy. The results concerning the impact of persistent pathological node involvement on outcome did not significantly different according to whether or not the patients had received preoperative radiotherapy (Table 5).

**Table 3** Long-term survival

| **(*n* = 487)** | **3 years (%)** | **95% CI** | **5 years (%)** | **95% CI** | **10 years (%)** | **95% CI** |
|---|---|---|---|---|---|---|
| Overall survival | 88.2 | 85.2–91.1 | 75.8 | 71.8–79.8 | 54.6 | 48.3–60.8 |
| Local relapse-free survival | 91.2 | 88.6–93.8 | 87.9 | 84.8–91.1 | 81.8 | 76.9–86.7 |
| Metastasis-free survival (median = 160 months) | 73.6 | 69.6–77.6 | 64.3 | 59.8–68.7 | 53.1 | 47.4–58.7 |
| Disease-free survival (median = 99 months) | 70.4 | 66.3–74.6 | 59.6 | 55–64.1 | 47.6 | 41.9–53.2 |

*© 2000 Cancer Research Campaign*

**Table 4** Survival according to lymph-node status

| Nodes | Patients | DFS median (months) | DFS 5 years ± SD | DFS 10 years ± SD | OS median (months) | OS 5 years ± SD | OS 10 years ± SD |
|---|---|---|---|---|---|---|---|
| Total | 487 (100%) | 99 | 60 ± 2% | 48 ± 3% | not reached | 76 ± 2% | 55 ± 3% |
| 0 | 223 (45.8%) | not reached | 72 ± 3% | 63 ± 4% | not reached | 83 ± 3% | 64 ± 5% |
| 1–3 | 159 (32.6%) | 85 | 58 ± 4% | 42 ± 5% | 125 | 76 ± 4% | 55 ± 5% |
| 4–7 | 72 (14.8%) | 55 | 47 ± 6% | 35 ± 7% | 91 | 68 ± 6% | 43 ± 8% |
| ≥ 8 | 34 (7%) | 20 | 15 ± 6% | 7 ± 5% | 48 | 44 ± 9% | 26 ± 8% |
| Logrank test | | | $P < 0.0001$ | | | $P < 0.0001$ | |

**Table 5** Disease-free survival (DFS) and overall survival (OS) according to lymph-node status and preoperative radiotherapy

| Nodes | 5-year DFS ± SD woPRT (*n* = 322) | 5-year DFS ± SD PRT (*n* = 166) | 5-year OS ± SD woPRT (*n* = 322) | 5-year OS ± SD PRT (*n* = 166) |
|---|---|---|---|---|
| 0 | 76.9 ± 6.8% | 60.1 ± 12.3% | 86.5 ± 5.9% | 75.4 ± 10.9% |
| 1–3 | 61.8 ± 10.2% | 52.1 ± 12.9% | 83.1 ± 7.8% | 66.6 ± 12.3% |
| 4–7 | 49.1 ± 15.9% | 44 ± 19.4% | 63.9 ± 15.5% | 53.1 ± 17.1% |
| ≥ 8 | 12.5 ± 13.3% | 20 ± 24.9% | 54.2 ± 19.9% | 20 ± 24.7% |

woPRT = without preoperative radiotherapy; PRT = with preoperative radio therapy

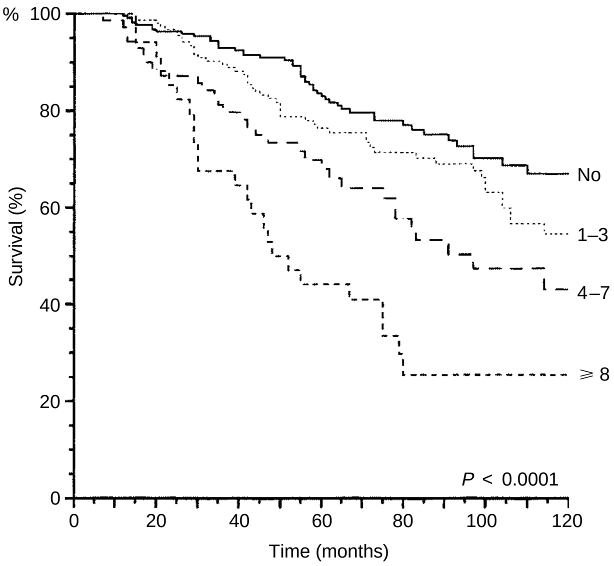

**Figure 2** Overall survival according to node involvement (*n* = 487)

The 5-year and 10-year survival rates for the complete 'reference group' (936 patients) were 80.4% (95% CI, 77.8–83.1) and 59.7% (95% CI, 55.7–63.7), respectively, and the corresponding 5-year and 10-year disease-free survival rates were 59.5% (95% CI, 56.4–62.6) and 45.8% (95% Cl, 42–49.6), respectively.

### Univariate and multivariate analysis

On univariate analysis, pathological node status was found to be correlated with both clinical lymph-node status before chemotherapy and clinical response of the primary tumour

**Table 6** Correlation of histological lymph-node status and other prognostic factors (univariate analysis)

| Patient characteristics | *n* | pN0 (*n* = 223) | pN1 (*n* = 265) | *P* |
|---|---|---|---|---|
| Clinical TUICC | | | | |
| T2 | 312 | 47.4% | 52.6% | |
| T3 | 176 | 42.6% | 57.4% | ns |
| Clinical NUICC | | | | |
| N0 N1a | 219 | 68.5% | 31.5% | < 0.0001 |
| N1b | 269 | 27.2% | 72.8% | |
| SBR Grade | | | | |
| I–II | 335 | 46.3% | 53.7% | |
| III | 88 | 43.2% | 56.8% | ns |
| unknown | 65 | | | |
| Age | | | | |
| ≤ 40 years | 107 | 42.9% | 57.1% | ns |
| > 40 years | 381 | 46.5% | 53.5% | |
| Histology | | | | |
| ductal | 420 | 44.7% | 55.3% | |
| lobular | 32 | 53.1% | 46.9% | ns |
| others | 30 | 53.4% | 46.6% | |
| unknown | 6 | | | |
| Oestrogen receptors | | | | |
| negative | 165 | 49.7% | 21.8% | ns |
| positive | 220 | 42.7% | 20.5% | |
| unknown | 103 | | | |
| Progesterone receptors | | | | |
| negative | 202 | 44.6% | 55.4% | ns |
| positive | 222 | 43.7% | 56.3% | |
| unknown | 64 | | | |
| S Phase | | | | |
| ≤ 5% | 132 | 49.2% | 50.8% | |
| > 5% | 93 | 50.5% | 49.5% | ns |
| unknown | 263 | | | |
| Objective clinical response | | | | |
| Yes | 255 | 50.2% | 49.8% | 0.04 |
| No | 232 | 40.8% | 59.2% | |
| Preoperative radiotherapy | | | | |
| Yes | 166 | 40.9% | 59.1% | |
| No | 321 | 48.0% | 52.0% | ns |

© 2000 Cancer Research Campaign

**Table 7** Clinical response to chemotherapy (univariate analysis)

| Characteristics | Objective clinical response | No response | *P* |
|---|---|---|---|
| TUICC | | | |
| T2 | 61.4% (191/311) | 38.6% (120/311) | |
| T3 | 36.4% (64/176) | 63.6% (112/176) | < 0.001 |
| Mean tumour size | 4.6 ± 1.5 cm | 5.3 ± 1.9 cm | < 0.001[a] |
| NUICC | | | |
| N0 N1a | 57.3% (125/218) | 42.7% (93/218) | |
| N1b | 48.3% (130/269) | 51.7% (139/269) | 0.05 |
| SBR Grade | | | |
| I–II | 51.5% (172/334) | 48.5% (162/334) | |
| III | 51.1% (45/88) | 48.9% (43/88) | ns |
| Age | | | |
| ≤ 40 years | 52.3% (56/107) | 47.7% (51/107) | |
| > 40 years | 52.4% (199/380) | 47.6% (181/380) | ns |
| Histology | | | |
| ductal | 52.8% (222/420) | 47.2% (198/420) | |
| lobular | 45.1% (14/31) | 54.9% (17/31) | |
| | 50% (15/30) | 50% (15/30) | ns |
| Oestrogen receptors | | | |
| negative | 58.2% (96/165) | 41.8% (69/165) | |
| positive | 49.3% (108/219) | 50.7% (111/219) | 0.08 |
| Pregesterone receptors | | | |
| negative | 58.2% (117/201) | 41.8% (84/201) | |
| positive | 46.4% (103/222) | 53.6% (119/222) | 0.01 |
| Histological lymph-node status | | | |
| pN0 | 57.4% (128/223) | 42.6% (95/223) | |
| pN1 | 48.2% (127/265) | 51.8% (138/265) | 0.04 |
| S Phase | | | |
| ≤ 5% | 53.8% (71/132) | 46.2% (61/132) | |
| > 5% | 67.8% (63/93) | 32.2% (30/93) | 0.03 |
| Preoperative radiotherapy | | | |
| Yes | 27.7% (46/166) | 72.7% (120/166) | |
| No | 65.1% (209/321) | 34.9% (112) | < 0.001 |

[a]Student *t*-test.

(Table 6). 27% of patients diagnosed to have positive nodes on clinical examination prior to treatment were node-negative on pathological examination after chemotherapy and surgery. Clinical response was correlated with small tumour size, negative clinical lymph-node status, absence of progesterone receptors and high S-phase values (Table 7). Univariate analysis also showed that all of the usual prognostic parameters assessed in this study were correlated with disease-free survival and overall survival (Table 8). No correlation was demonstrated between histological response and survival, probably because pathological response was obtained in only 5% of patients. In a multivariate model (Table 9), outcome remained strongly correlated with pathological lymph-node status, with a 4.3-fold increased relative risk of death for patients with eight or more positive nodes. Tumour grade and hormonal receptor status were also associated with survival and disease-free survival. Young age was associated with a short disease-free survival. Preoperative radiotherapy was performed predominantly in poor responders to chemotherapy (72.7%) and therefore is associated to a poorer outcome in the univariate analysis for survival. On the multivariate analysis, the clinical response of the tumour was found to be an independent prognostic value of survival and preoperative radiotherapy had no more prognostic significance.

### DISCUSSION

The increase in the number of positive nodes is almost linearly correlated with a decline in survival and the potential to achieve cure (Fisher et al, 1983). The prognostic value of persistent node involvement following neoadjuvant chemotherapy for locally advanced breast cancer has been evaluated in several studies (McCready et al, 1989; Gardin et al, 1995; Machiavelli et al, 1998; Kuerer et al, 1999*a*). Fewer reports have been published in operable breast cancer. Ellis et al (1998) showed that clinical but not pathological axillary-node status was a major predictor of outcome following primary chemotherapy. Conversely, pathological nodal status was reported by Bonadonna et al, 1998) and Cameron et al, (1997) to be the major prognostic factor associated with clinical response to treatment on multivariate analysis. In another smaller series, it was the only prognostic factor identified by a multivariate model (Botti et al, 1995). Data from the MD Anderson Hospital reported similar 10-year survival rates for primary doxorubicin-based chemotherapy. Women with stage III breast cancer had survival rates of 65%, 44%, 32% and 9%, when zero, 1–3, 4–9, and 10 nodes were still involved after chemotherapy, respectively (Frye et al, 1995).

It could be argued that the prognostic significance of the number of nodes still containing tumour after preoperative chemotherapy might simply reflect the number of nodes involved at presentation. However, in the NSABP B18 trial, a 37% increase in the incidence of pathologically negative nodes was seen following preoperative chemotherapy: 43% in the postoperative group compared to 59% in the preoperative group (Fisher et al, 1997). This decrease in pathological nodal involvement was equally distributed in all categories of node involvement (1–3, 4–9 or ≥ 10 positive nodes). However, it is not possible to separately identify those patients in whom all histologically involved nodes were cured by preoperative treatment. In our study, pathological nodal status was significantly correlated with clinical response ($P = 0.04$). The absence of involved nodes may therefore reflect drug sensitivity, at least in some cases, and Cox multivariate analysis revealed this factor to be a more sensitive marker than clinical tumour response.

Clinical response was an independent prognostic factor for survival in this study, as previously reported in other trials performed both in our institution (Scholl et al 1995) and by others (Cameron et al, 1997). In the present study, radiotherapy alone with a radiation boost to the tumour bed was proposed as sole local – regional treatment for very good responders. Consequently, no information was available about pathological axillary lymph-node status in these patients. Pathological tumour response has been reported to be a more powerful prognostic factor than clinical response in locally advanced breast cancer (Sataloff et al, 1995; Kuerer et al, 1999*b*) and more recently in operable breast cancer. The pathological complete response rate including pathological lymph-node status was 9% in the NSABP-B18 trial (Fisher et al, 1998) and quite low in the Milan experience (3%) (Bonadonna et al, 1998).

The persistence of nodal involvement after neo-adjuvant therapy may represent the existence of persistent systemic micrometastases which are ultimately responsible for the patient’s demise. However, in the present study, one might expect that patients also given loco–regional radiotherapy would have less nodal involvement for the same degree of micrometastatic disease. The systemic disease had been exposed to identical anti-cancer therapy, but the local nodes would have received radiotherapy. There is a trend to a worse survival for patients who received preoperative radiotherapy for the same number of involved nodes. However, statistical comparisons were not significant.

*© 2000 Cancer Research Campaign*

**Table 8** Disease-free survival (DFS) and overall survival (univariate analysis, *n* = 487)

| Characteristics | *n* | DFS 5 years (%) | DFS 10 years (%) | Logrank | Overall survival 5 years (%) | Overall survival 10 years (%) | Logrank |
|---|---|---|---|---|---|---|---|
| TUICC | | | | | | | |
| T2 | 311 | 62.3 | 45.7 | | 80.9 | 58.7 | |
| T3 | 176 | 54.9 | 49.1 | *P* = 0.33 | 67.4 | 47.7 | *P* = 0.01 |
| NUICC | | | | | | | |
| N0 N1a | 218 | 71 | 59 | | 83.2 | 63.3 | |
| N1b | 269 | 51 | 39 | *P* < 0.0001 | 70.1 | 49.1 | *P* = 0.0012 |
| Age | | | | | | | |
| ≤ 40 years | 107 | 48 | 33 | | 69 | 48 | |
| > 40 years | 380 | 63 | 53 | *P* = 0.0004 | 78 | 57 | *P* = 0.06 |
| SBR Grade | | | | | | | |
| I–II | 334 | 63 | 51 | | 79.7 | 62 | |
| III | 88 | 47.7 | 36 | *P* = 0.001 | 56.5 | 35.9 | *P* < 0.0001 |
| Histology | | | | | | | |
| Ductal | 419 | 57.3 | 43.8 | | 74.9 | 52.2 | |
| Lobular | 32 | 75.8 | 55.6 | ns | 86.9 | 81.8 | ns |
| Progesterone receptors | | | | | | | |
| negative | 201 | 52 | 39 | | 66 | 41 | |
| positive | 221 | 65 | 52 | *P* = 0.005 | 83 | 65 | *P* < 0.0001 |
| Oestrogen receptors | | | | | | | |
| negative | 165 | 55 | 39 | | 68 | 49 | |
| positive | 218 | 64 | 47 | ns | 82 | 57 | *P* = 0.02 |
| Node involvement | | | | | | | |
| pN0 | 223 | 71.8 | 63.3 | | 83.1 | 64.4 | |
| pN 1–3 | 158 | 57.8 | 42.3 | | 76.4 | 54.6 | |
| pN 4–7 | 72 | 47.5 | 34.5 | | 68.1 | 43.1 | |
| pN ≥ 8 | 34 | 14.7 | 7.3 | *P* < 0.0001 | 44.1 | 25.5 | *P* < 0.0001 |
| Clinical response | | | | | | | |
| Yes | 255 | 65.1 | 49.9 | | 79.1 | 63.1 | |
| No | 231 | 53.3 | 44.7 | *P* = 0.04 | 72.1 | 47.2 | *P* = 0.01 |
| Preoperative radiotherapy | | | | | | | |
| Yes | 166 | 52.1 | 42.1 | | 68.3 | 46.8 | |
| No | 321 | 63.5 | 50.4 | *P* = 0.04 | 79.9 | 59.9 | *P* = 0.004 |
| S Phase | | | | | | | |
| ≤ 5% | 131 | 64.4 | 53.1 | | 86.6 | 53.3 | |
| > 5% | 93 | 51.1 | 24.8 | *P* = 0.009 | 66.7 | 38.4 | *P* = 0.009 |

Prediction of response is important, as response to treatment constitutes a major prognostic factor. In the present study, tumour response was also related to tumour size and clinical tumour shrinkage was more marked in smaller tumours, as also reported in the Milan series (Bonadonna et al, 1998). The probability of axillary lymph-node involvement has been reported to progressively increase with increasing size of the tumour (Nemoto et al, 1980), but this relation was not observed in our study, possibly because only T2 and T3 tumours were analysed. In our experience, absent progesterone receptor (PR) expression correlated favourably with response to chemotherapy and unfavourably with survival. Colleoni had previously reported this correlation in a series of 73 patients receiving neoadjuvant chemotherapy (Colleoni et al, 1999). MacGrogan detected significant chemosensitivity for ER-negative tumours, but not for PR-negative tumours (MacGrogan et al, 1996), whereas other authors failed to observe a correlation between hormone-receptor expression and response to chemotherapy (Jain et al, 1996; Makris et al, 1997). One hypothesis could be that PR-negative tumours have high proliferation, since less differentiated. Therefore they relapse more rapidly but are equally more sensitive to chemotherapy. In more recent publications, patient selection according to biological predictive factors for response, including high S phase and c-erbB-2 overexpression, remains one of the most challenging issues in neoadjuvant chemotherapy (Colleoni et al, 1999). S phase was available in only a small fraction of the patients in this series. Nevertheless, high S phase remained predictive of clinical response to neoadjuvant chemotherapy on multivariate analysis as previously reported (Remvikos et al, 1989). Pathological lymph-node involvement may constitute a response criterion to determine the efficacy of neoadjuvant chemotherapy. The correlation between the risk of developing distant metastases and axillary content is consistent with the hypothesis that axillary involvement is an index of the capacity for tumour spread, but it is not the cause of dissemination and may not be a predictive marker of response to treatment.

How can the efficacy of neoadjuvant chemotherapy be increased? Complete clinical response rates of more than 60% have been obtained following continuous infusion of 5-fluorouracil associated with epirubicin and cisplatin (Smith et al, 1995). New drugs such as taxanes are currently under investigation in combination with anthracyclines as neoadjuvant treatment in order to increase the pathological complete response rate (Costa et al, 1999). The role of docetaxel is being investigated before or after neoadjuvant chemotherapy in the current NSABP trial B27 (Mamounas, 1998). One of the secondary objectives of this trial is to determine whether there might be a benefit from addition of postoperative docetaxel chemotherapy, particularly in a subgroup of patients such as those with residual positive nodes after a

*© 2000 Cancer Research Campaign* 

**Table 9** Multivariate analysis (*n* = 487)

| | Disease free survival | | | | Overall survival | | |
|---|---|---|---|---|---|---|---|
| | ***n*** | **RR** | **95% CI** | ***P*** | **RR** | **95% CI** | ***P*** |
| Node involvement | | | | | | | |
| None | 222 | 1 | – | | 1 | – | |
| 1–3 | 159 | 1.6 | 1.2–2.3 | | 1.3 | 0.9–1.9 | |
| 4–7 | 72 | 2.3 | 1.5–3.4 | < 0.001 | 1.9 | 1.2–3.1 | < 0.0001 |
| ≥8 | 34 | 6.3 | 4.1–9.7 | | 4.3 | 2.6–7.1 | |
| SBR Grade | | | | | | | |
| I–II | 333 | 1 | – | | 1 | – | |
| III | 88 | 1.6 | 1.2–2.2 | < 0.01 | 1.9 | 1.4–2.9 | 0.005 |
| Pregesterone receptors | | | | | | | |
| positive | 221 | 1 | – | | 1 | – | |
| negative | 201 | 1.4 | 1.1–2 | < 0.05 | 2 | 1.4–3.3 | < 0.01 |
| Response | | | | | | | |
| Yes | 255 | – | | 1 | – | | |
| No | 232 | – | ns | 1.5 | 1.1–2.1 | 0.01 | |
| Age | | | | | | | |
| >40 years | 380 | 1 | – | | – | | |
| ≤40 years | 107 | 1.4 | 1.1–1.9 | <0.05 | | – | ns |

preoperative combination of doxorubicin and cyclophosphamide. The Aberdeen Breast Group has reported the activity of neo-adjuvant docetaxel in patients with a poor response to anthracyline-based chemotherapy (Hutcheon et al, 2000). Other second-line treatments with or without high-dose chemotherapy might also be considered in this population. Although recent studies have reported disappointing results of chemotherapy intensification (Pusztai and Hortobagyi, 1998), we feel that this strategy should be investigated in patients with a high S phase.

Although preoperative tumour debulking is considered to be a favourable prognostic factor, there is still a controversy concerning the need to perform axillary dissection following an excellent clinical response to systemic induction therapy. According to the results of the NSABP B18 trial, 26% of clinically node-negative patients with tumours ≤ 2 cm had pathologically positive nodes after preoperative chemotherapy (Fisher et al, 1997). Although preoperative therapy induces downstaging of axillary lymph-node status, persistent pathological node involvement is an unfavourable prognostic factor and does not argue in favour of elimination of axillary lymph-node dissection. Since the use of sentinel lymph-node biopsy as an alternative to axillary dissection is becoming increasingly popular (Krag et al, 1998), this procedure might be further investigated in patients receiving neoadjuvant chemotherapy (Kuerer et al, 1999*b*). Patients with locally advanced breast cancer and clinically positive axillary lymph nodes following neoadjuvant chemotherapy will benefit from axillary dissection to ensure local control (Kuerer et al, 1998). However, the benefit of axillary dissection in patients with a clinically negative axilla may be minimal and potentially harmful if the axilla is irradiated, particularly as pathological staging does not affect subsequent systemic treatment. A prospective randomized trial of axillary dissection vs axillary radiotherapy in patients with a clinically negative axilla following neoadjuvant chemotherapy is presently underway to evaluate this hypothesis (Kuerer et al, 1998).

In conclusion, pathological lymph-node status after neoadjuvant chemotherapy remains the major prognostic factor for survival in large operable breast cancers. Axillary lymph-node dissection should be considered to be an important component of combined modality therapy for patients with large resectable breast carcinoma, in order to identify subgroups of patients who may benefit from alternative treatments in the adjuvant setting. New strategies must be designed to increase the clinical response rate and pathological tumour sterilization rate. The poor prognosis of patients with extensive lymph-node involvement after preoperative therapy justifies the development of alternative treatment modalities.

### REFERENCES

Bhalla K and Harris WB (1998) Molecular and biologic determinants of neoadjuvant chemotherapy of locoregional breast cancer. *Semin Oncol* **25** (suppl 3): 19–24

Bonadonna G, Valagussa P, Brambilla C, Ferrari L, Moliterni A, Terenziani M and Zambetti M (1998) Primary chemotherapy in operable breast cancer: eight-year experience at the Milan Cancer Institute. *J Clin Oncol* **16**: 93–100

Botti C, Vici P, Lopez M, Scinto AF, Cognetti F and Cavaliere R (1995) Prognostic value of lymph node metastases after neoadjuvant chemotherapy for large-sized operable carcinoma of the breast. *J Am Coll Surg* **181**: 202–208

Cameron DA, Anderson ED, Levack P, Hawkins RA, Anderson TJ, Leonard RC, Forrest AP and Chetty U (1997) Primary systemic therapy for operable breast cancer – 10-year survival data after chemotherapy and hormone therapy. *Br J Cancer* **76**: 1099–1105

Carter CL, Allen C and Henson DE (1989) Relation of tumor size, lymph node status, and survival in 24,740 breast cancer cases. *Cancer* **63**: 181–187

Colleoni M, Orvieto E, Nolé F and Orlando L (1999) Prediction of response to primary chemotherapy for operable breast cancer. *Eur J Cancer* **35**: 574–579.

Costa SD, von Minckwitz G, Raab G, Blohmer JU, Dresel V, Eidtmann H, Hilfrich J, Jackisch C, Merkle E, Gademann G and Kaufmann M (1999) The role of docetaxel (Taxotere) in neoadjuvant chemotherapy of breast cancer. *Semin Oncol* **26**: 24–31

Cox DR (1972) Regression models and life tables (with discussion). *J Stat Soc B* **34**: 187–220

Ellis P, Smith I, Ashley S, Walsh G, Ebbs S, Baum M, Sacks N and McKinna J (1998) Clinical prognostic and predictive factors for primary chemotherapy in operable breast cancer. *J Clin Oncol* **16**: 107–114

Fisher B, Bauer M, Wickerham DL, Redmond CK, Fisher ER, Cruz AB, Foster R, Gardner B, Lerner H, Margolese R, Poisson R, Shibata H and Volk H (1983) Relation of number of positive axillary nodes to the prognosis of patients with primary breast cancer. An NSABP update. *Cancer* **52**: 1551–7

Fisher B, Brown A, Mamounas E, Wieand S, Robidoux A, Margolese RG, Cruz AB, Jr, Fisher ER, Wickerham DL, Wolmark N, DeCillis A, Hoehn JL, Lees AW and Dimitrov NV (1997) Effect of preoperative chemotherapy on local-regional disease in women with operable breast cancer: findings from National Surgical Adjuvant Breast and Bowel Project B-18. *J Clin Oncol* **15**: 2483–2493

Fisher B, Bryant J, Wolmark N, Mamounas E, Brown A, Fisher ER, Wickerham DL, Begovic M, DeCillis A, Robidoux A, Margolese RG, Cruz AB, Jr, Hoehn JL,

 *© 2000 Cancer Research Campaign*

Lees AW, Dimitrov NV and Bear HD (1998) Effect of preoperative chemotherapy on the outcome of women with operable breast cancer. *J Clin Oncol* **16**: 2672–2685

Frye D, Buzdar A and Hortobagyi G (1995) Prognostic significance of axillary involvement after preoperative chemotherapy in stage III breast cancer. *Proc Am Soc Clin Oncol* **14**: 95 (abst 81)

Gardin G, Rosso R, Campora E, Repetto L, Naso C, Canavese G, Catturich A, Corvo R, Guenzi M, Pronzato P, Baldini E and Conte PF (1995) Locally advanced non-metastatic breast cancer: analysis of prognostic factors in 125 patients homogeneously treated with a combined modality approach. *Eur J Cancer* **31A**: 1428–1433

Haagensen CD (1977) Treatment of curable carcinoma of the breast. *Int J Radiat Oncol Biol Phys* **2**: 975–980

Hutcheon AW, Smith IC, Heys SD, Ogston KN, Miller ID, Payne S, Eremin O, Ah-See AK, Eggleton P and Group TAB (2000) Primary chemotherapy in breast cancer: significantly enhanced clinical and pathological response with docetaxel. *Proc Am Soc Clin Oncol* **19**: (Abst 317)

Jain V, Landry M and Levine EA (1996) The stability of estrogen and progesterone receptors in patients receiving preoperative chemotherapy for locally advanced breast carcinoma. *Am Surg* **62**: 162–165

Kaplan EL and Meier P (1958) Nonparametric estimation from incomplete observations. *J Am Stat Assoc* **53**: 457–481

Krag D, Weaver D, Ashikaga T, Moffat F, Klimberg VS, Shriver C, Feldman S, Kusminsky R, Gadd M, Kuhn J, Harlow S and Beitsch P (1998) The sentinel node in breast cancer – a multicenter validation study. *N Engl J Med* **339**: 941–946

Kuerer HM, Newman LA, Buzdar AU, Hunt KK, Dhingra K, Buchholz TA, Binkley SM, Ames FC, Feig BW, Ross MI, Hortobagyi GN and Singletary SE (1998) Residual metastatic axillary lymph nodes following neoadjuvant chemotherapy predict disease-free survival in patients with locally advanced breast cancer. *Am J Surg* **176**: 502–509

Kuerer HM, Newman LA, Smith TL, Ames FC, Hunt KK, Dhingra K, Theriault RL, Singh G, Binkley SM, Sneige N, Buchholz TA, Ross MI, McNeese MD, Buzdar AU, Hortobagyi GN and Singletary SE (1999*a*) Clinical course of breast cancer patients with complete pathologic primary tumor and axillary lymph node response to doxorubicin-based neoadjuvant chemotherapy [see comments]. *J Clin Oncol* **17**: 460–469

Kuerer HM, Sahin AA, Hunt KK, Newman LA, Breslin TM, Ames FC, Ross MI, Buzdar AU, Hortobagyi GN and Singletary SE (1999*b*) Incidence and impact of documented eradication of breast cancer axillary lymph node metastases before surgery in patients treated with neoadjuvant chemotherapy. *Ann Surg* **230**: 72–78

MacGrogan G, Mauriac L, Durand M, Bonichon F, Trojani M, de Mascarel I and Coindre JM (1996) Primary chemotherapy in breast invasive carcinoma: predictive value of the immunohistochemical detection of hormonal receptors, p53, c-erbB-2, MiB1, pS2 and GST pi. *Br J Cancer* **74**: 1458–1465

Machiavelli MR, Romero AO, Perez JE, Lacava JA, Dominguez ME, Rodriguez R, Barbieri MR, Romero Acuna LA, Romero Acuna JM, Langhi MJ, Amato S, Ortiz EH, Vallejo CT and Leone BA (1998) Prognostic significance of pathological response of primary tumor and metastatic axillary lymph nodes after neoadjuvant chemotherapy for locally advanced breast carcinoma. *Cancer J Sci Am* **4**: 125–131

Makris A, Powles TJ, Dowsett M, Osborne CK, Trott PA, Fernando IN, Ashley SE, Ormerod MG, Titley JC, Gregory RK and Allred DC (1997) Prediction of response to neoadjuvant chemoendocrine therapy in primary breast carcinomas. *Clin Cancer Res* **3**: 593–600

Mamounas EP (1998) Overview of National Surgical Adjuvant Breast Project neoadjuvant chemotherapy studies. *Semin Oncol* **25**: 31–35

Mantel N (1966) Evaluation of survival data and two new rank order statistics arising in its consideration. *Cancer Chemother Rep*, **50**: 163–170

Mauriac L, MacGrogan G, Avril A, Durand M, Floquet A, Debled M, Dilhuydy JM and Bonichon F (1999) Neoadjuvant chemotherapy for operable breast carcinoma larger than 3 cm: a unicentre randomized trial with a 124-month median follow-up. Institut Bergonie Bordeaux Groupe Sein (IBBGS). *Ann Oncol*, **10**: 47–52

McCready DR, Hortobagyi GN, Kau SW, Smith TL, Buzdar AU and Balch CM (1989) The prognostic significance of lymph node metastases after preoperative chemotherapy for locally advanced breast cancer. *Arch Surg* **124**: 21–225

Nemoto T, Vana J, Bedwani RN, Baker HW, McGregor FH and Murphy GP (1980) Management and survival of female breast cancer: results of a national survey by the American College of Surgeons. *Cancer* **45**: 2917–2924

Oken MM, Creech RH, Tormey DC, Horton J, Davis TE, McFadden ET and Carbone PP (1982) Toxicity and response criteria of the Eastern Cooperative Oncology Group. *Am J Clin Oncol* **5**: 649–655

Powles TJ, Hickish TF, Makris A, Ashley SE, O'Brien ME, Tidy VA, Casey S, Nash AG, Sacks N, Cosgrove D, MacVicar D, Fernando I and Ford HT (1995) Randomized trial of chemoendocrine therapy started before or after surgery for treatment of primary breast cancer. *J Clin Oncol* **13**: 547–552

Pusztai L and Hortobagyi GN (1998) Discouraging news for high-dose chemotherapy in high-risk breast cancer. *Lancet* **352**: 501–502

Remvikos Y, Beuzeboc P, Zajdela A, Voillemot N, Magdelenat H and Pouillart P (1989) Correlation of pretreatment proliferative activity of breast cancer with the response to cytotoxic chemotherapy. *J Natl Cancer Inst* **81**: 1383–1387

Remvikos Y, Mosseri V, Zajdela A, Fourquet A, Durand JC, Pouillart P and Magdelenat H (1993) Prognostic value of the S-phase fraction of breast cancers treated by primary radiotherapy or neoadjuvant chemotherapy. *Ann N Y Acad Sci* **698**: 193–203

Sataloff DM, Mason BA, Prestipino AJ, Seinige UL, Lieber CP and Baloch Z (1995) Pathologic response to induction chemotherapy in locally advanced carcinoma of the breast: a determinant of outcome. *J Am Coll Surg* **180**: 297–306

Scholl SM, Fourquet A, Asselain B, Pierga JY, Vilcoq JR, Durand JC and Pouillart P (1994) Neoadjuvant versus adjuvant chemotherapy in premenopausal patients with tumours considered too large for breast conserving surgery: preliminary results of a randomised trial: S6. *Eur J Cancer* **30A**: 645–652

Scholl SM, Pierga JY, Asselain B, Beuzeboc P, Dorval T, Garcia-Giralt E, Jouve M, Palangie T, Remvikos Y, Durand JC, Fourquet A and Pouillart P (1995) Breast tumour response to primary chemotherapy predicts local and distant control as well as survival. *Eur J Cancer* **31A**: 1969–1975

Semiglazov VF, Topuzov EE, Bavli JL, Moiseyenko VM, Ivanova OA, Seleznev IK, Orlov AA, Barash NY, Golubeva OM and Chepic OF (1994) Primary (neoadjuvant) chemotherapy and radiotherapy compared with primary radiotherapy alone in stage IIb-IIIa breast cancer. *Ann Oncol* **5**: 591–595

Smith IE, Walsh G, Jones A, Prendiville J, Johnston S, Gusterson B, Ramage F, Robertshaw H, Sacks N, Ebbs S, McKinna JA and Baum M (1995) High complete remission rates with primary neoadjuvant infusional chemotherapy for large early breast cancer. *J Clin Oncol* **13**: 424–429

Valagussa P, Bonadonna G and Veronesi U (1978) Patterns of relapse and survival following radical mastectomy. Analysis of 716 consecutive patients. *Cancer* **41**: 1170–118

*© 2000 Cancer Research Campaign* 
